# Feasibility of photoacoustic/ultrasound imaging of synovitis in finger joints using a point-of-care system

**DOI:** 10.1016/j.pacs.2017.08.002

**Published:** 2017-08-31

**Authors:** Pim J. van den Berg, Khalid Daoudi, Hein J. Bernelot Moens, Wiendelt Steenbergen

**Affiliations:** a Biomedical Photonic Imaging, MIRA Institute for Biomedical Technology and Technical Medicine, University of Twente, PO Box 217, 7500 AE, Enschede, The Netherlands; b Medical Ultrasound Imaging Center, department of Radiology, Radboud University Medical Center, PO Box 9101, 6500 HB Nijmegen, The Netherlands; c Ziekenhuisgroep Twente, Department of Rheumatology, Postbus 546, 7550 AM Hengelo, The Netherlands

**Keywords:** Rheumatoid arthritis, Synovitis, Photoacoustic, Optoacoustic, Medical ultrasound, Echography, Ultrasonography, Proof of principle, Feasibility study

## Abstract

We evaluate a portable ultrasound and photoacoustic imaging (PAI) system for the feasibility of a point-of-care assessment of clinically evident synovitis. Inflamed and non-inflamed proximal interphalangeal joints of 10 patients were examined and compared with joints from 7 healthy volunteers. PAI scans, ultrasound power Doppler (US-PD), and clinical examination were performed. We quantified the amount of photoacoustic (PA) signal using a region of interest (ROI) drawn over the hypertrophic joint space. PAI response was increased 4 to 10 fold when comparing inflamed with contralateral non-inflamed joints and with joints from healthy volunteers (p < 0.001 for both). US-PD and PAI were strongly correlated (Spearman’s ρ = 0.64, with 95% CI: 0.42, 0.79). Hence, PAI using a compact handheld probe is capable of detecting clinically evident synovitis. This motivates further investigation into the predictive value of PAI, including multispectral PAI, with other established modalities such as US-PD or MRI.

## 1 Introduction

In rheumatoid arthritis (RA), imaging of synovitis with ultrasound power Doppler (US-PD) and magnetic resonance imaging (MRI) can predict disease progression and bone erosion [1], [2], [3]. In clinical remission, detection of subclinical synovitis indicates disease progression and increases the risk of disease flare [4], [5], [6], [7]. US-PD has gained a place in the clinical workflow based on these qualities. However, US-PD has inherently high operator dependency and suboptimal reproducibility [8], [9]. Specific complications of US-PD are its dependency on the angle between the flow vector and the sound beam, and the disturbance of the blood flow by the probe pressure. MRI is rather costly, specificity is modest and it requires contrast agents [10]. Optical imaging methods were studied in recent years as potential alternatives. Optical spectral transmission (OST) for example, has shown fair performance at detecting synovitis while being presumably low in cost [11], [12], [13], however, sensitivity and specificity are modest and the low spatial resolution limits differentiation between synovitis and tenosynovitis. Fluorescence optical imaging [14], [15], [16], [17] appears to have higher performance than OST, but also has low resolution and in addition requires injection of contrast agents.

Photoacoustic imaging (PAI), a hybrid optical-and-ultrasound imaging technique, may offer a good balance in features, combining the sensitivity to haemoglobin of optical techniques with the resolution of clinical ultrasound [18], [19], [20], [21]. To form a PA image, short laser pulses are shone on the skin and subsequently enter the tissue, where the light is scattered by cells and becomes diffuse. The light pulse is then absorbed by dark tissue constituents such as haemoglobin and melanin. The absorption slightly heats structures containing these constituents which leads to a small pressure build-up, generating sound waves that can be picked up by ultrasound transducers. PAI is therefore similar to sonography, except that the ultrasound is generated within tissue, instead of reflected ('backscattered') by it.

PAI differs significantly from US-PD in three aspects. First, movement of erythrocytes is not required for signal generation, since the generation of PA signals relies only on the presence of haemoglobin (or other chromophores) [19]. Second, there is a larger concentration of haemoglobin within vasculature than in surrounding tissue, leading to more signal generation, whereas in US-PD, erythrocytes reflect comparatively *less* signal than the surrounding tissue [22], [23]. A wall filter is therefore not required in PAI, and ‘flash' artefacts or motion clutter are not present. These properties imply that slow blood flow in synovial microvasculature poses no problem to PAI. As a result, we expect PAI to be particularly sensitive to subclinical synovitis. Finally, the PAI signal is less affected by the orientation of the blood vessel than US-PD.

PAI has been investigated in other medical areas involving angiogenesis, for instance in clinical studies into mammography [24], [25], [26], [27]. PAI has also been investigated in pre-clinical studies of synovitis [28], [29], [30], [31], and several setups have been proposed for human finger joints [32], [33], [34], [35], [36], [37]. In addition, a few early feasibility studies have been performed with RA patients [38], [39]. However, these studies used large lasers, not suited for routine clinical application, let alone point-of-care imaging.

In order to bring PAI to outpatient clinics, a handheld PA/US probe was developed [33], which in this study is investigated for possible use in assessing synovitis. The objective of this study is to investigate whether this PA/US probe can detect clinically evident synovitis and to compare the results with US-PD.

## 2 Methods

### 2.1 Patient inclusion

Patients undergoing care in the Ziekenhuisgroep Twente hospital were asked by their rheumatologist to participate in this study. Healthy volunteers were recruited in person or via flyers at the University of Twente.

Patients aged over 18 years with rheumatoid arthritis fulfilling 6 or more ACR/EULAR criteria (ACR/EULAR = American College of Rheumatology/European League Against Rheumatism) were included [40]. Specific inclusion criteria were: swelling of at least one proximal interphalangeal (PIP) joint, 2, 3 or 4 joints with at least grade 1 power-Doppler signal on US examination. Test subjects (healthy or patient) were excluded from participation if they had clinically significant bone deformation and/or osteoarthritis in the joint of interest. All subjects received written information and gave informed consent, resulting in a delay of 3 to 8 days between the inclusion by a rheumatologist and time of measurement.

### 2.2 Imaging system

**Fig. 1:**
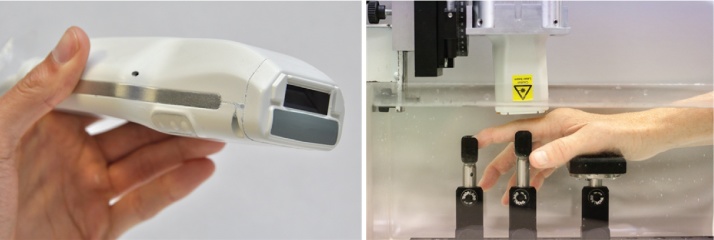
The PA/US probe (left) with view of the front end showing the light delivery window (dark aperture) and acoustic lens in medium gray. The patient’s hand is submerged in water (right) where it rests on a series of supports. The probe is mounted on a 2-axis motorized stage and positioned above the joint.

The imaging study is performed using a dual modality photoacoustic/ultrasound system. The system relies on a probe that houses both a small diode laser together with ultrasound transducers (see Fig. 1). The diode laser is pulsed to generate photoacoustic waves, which are then detected by the ultrasound transducers. These transducers are also used to transmit ultrasound to generate high-quality b-mode ultrasound images. The probe in this study is a second generation prototype developed from the probe described earlier in detail [33]. The original probe contained diode lasers producing 130 ns pulses at a 805 nm wavelength and a pulse energy of 0.56 mJ. As will appear, the main change is a doubling of the pulse energy.

The diode laser source (Quantel Laser, les Ulis, France) is controlled by a short pulse laser driver (Brightloop Converters, Paris, France) and generates 1 mJ pulses of 120 ns duration. The pulses are formed into a rectangular shape of 2.2 mm by 17.6 mm (1/e^2^) by a diffractive optical element (SILIOS Technologies, Peynier, France), after which the light exits the probe under an angle via a prism. The laser emission is at 808 nm, which corresponds to the isosbestic point of oxy-haemoglobin and deoxy-haemoglobin, which leads to PA signal amplitudes independent of the blood oxygenation.

The ultrasound detection is based on an ESAOTE SL3323 probe. Transducers are placed in an array of 128 elements. Each element has a bandwidth from 2.5 MHz to 10 MHz with a 7.5 MHz centre frequency. An acoustic lens (focal length: 24 mm) is placed in front of the transducers to moderately focus the detection in the elevational plane.

The probe is connected to a MylabOne ultrasound scanner (ESAOTE Europe), which can be used in two modes. In the first it transfers the collected time-pressure data from the middle 64 elements directly to a laptop. This mode is used to acquire photoacoustic data. In the second mode the scanner operates regularly and is used to acquire b-mode ultrasound using all 128 elements in a line-by-line transmission and acquisition scheme.

The US-PD examination is done using an identical MylabOne scanner (in the second mode as described above) in combination with a 14 MHz centre frequency linear array (SL3116, ESAOTE). The PRF was set at 750 Hz, and the wall filter at its lowest and the sensitivity at its highest setting.

### 2.3 Scan protocol

Per subject examination, a minimum of two PIP joints were scanned: one clinically inflamed joint and an uninflamed joint – preferably the same joint contra-lateral. A complete examination of one subject included a series of longitudinal images using power Doppler ultrasound for each applicable joint and another series using the PA/US system. Both examinations took place with the subject’s arm placed in a water bath fitted with supports for the arm, hand and the finger to be scanned (see Fig. 1). The water temperature was controlled to 29–31 °C during the examination. During measurements there was no contact of the PA/US and US-PD probes with the skin in order to avoid pressure artefacts. In addition, the PA/US probe was placed 4–5 mm from the skin such that the laser beam intersects with the ultrasound elevational plane at the skin surface.

For the PA/US examination the PA/US probe was placed on a motorized stage for better control of the measurement. The probe was aligned longitudinal to the finger and on the dorsal side. The stage was moved orthogonal to the finger in 0.5 mm steps for over 6 mm. At each step, a PA image was accumulated over 500 laser pulses for 0.25 s. Taking into account the angle of incidence of 52° with the orthogonal on the skin and the beam size of 2.2 mm by 17.6 mm, the light exposure is 3.2 mW/cm^2^, which is below the IEC 60825-1 safety limit of 5 mW/cm^2^ for this wavelength and pulse train. In addition, 100 frames of plane wave ultrasound (one fixed angle) were recorded each step. Each scan was repeated with the same probe and at identical steps, but then with high-quality line-by-line b-mode ultrasound. One scan yielded therefore 13 PA, 13 plane wave and 13 b-mode images at identical locations. In our scan protocol there was approximately 1 min between a PA acquisition and the subsequent b-mode US image.

US-PD examination was either performed by an experienced rheumatologist or by placing the US-PD probe in the motorized stage. For each joint, 3–5 images are recorded.

### 2.4 Scoring of US-PD images

Representative US-PD images were digitally stored and anonymized. They were graded (0–3) according to Szkudlarek et al. [40] by two rheumatologists who were blinded to the allocation of the images. The widely used semi-quantitative grading system is based on visual assessment of blood flow as indicated by power-Doppler signals: no signals (score 0), up to 3 single vessel signals (score 1), confluent vessel signals in less than half of the area of the synovium (2) or vessel signals in more than half of the area of the synovium (3). Discrepant results were reviewed to reach consensus resulting in a final PD-score for each individual joint.

### 2.5 Data analysis

The PA channel data – the pressure as a function of time as measured by the transducers – is converted into a map of the original pressure distribution using a Fourier domain reconstruction algorithm [41]. For this reconstruction algorithm, we found an axial resolution of 0.2 mm and a lateral resolution of 0.4 mm [33]. The algorithm was selected for its computational speed. All data analysis is automated using Matlab (Massachusetts, USA). To account for the light attenuation within tissue, a depth-dependent correction ('gain') is applied. Since the finger in the longitudinal orientation is fairly flat, a basic exponential gain of $1/\text{exp}\left(-\mu_{\text{eff}}z\right)$ is used with *μ*_eff_ = 1/mm the effective attenuation coefficient and z the depth in tissue [42], [43]. A different *z* *=* *0* is set for every axial line in the PA image, such that the fluence correction starts at the skin level. Determining the position of the skin surface was done visually using the PA response from the melanin layer in the skin.

For image formation, the PA data is compressed logarithmically at a dynamic range of 40 dB or 18 dB, with the same minimum and maximum amplitude for inflamed and non-inflamed images. These dynamic ranges were selected based on the noise level (−40 dB) and the amplitude of healthy joint’s background PA signals (−18 dB) respectively. Pixels within the dynamic range are color coded in Matlab’s red-and-yellow color map ‘hot’ and finally overlaid on a b-mode ultrasound image.

For each joint scan, a region-of-interest (ROI) is drawn to select the hypertrophic joint area. The ROI is drawn on the b-mode ultrasound image, where the hypertrophic area is defined as to include any pixels between the tendon and the bone surface. The ROI is then transferred to the PA image, from which the number of PA pixels is calculated that fall within the 18 dB dynamic range. A secondary quantification metric is provided by the mean amplitude of non-compressed PA signals within the ROI. In case of healthy joints there is no hypertrophic area and the ROI selection will include more tissues than just the synovial space.

### 2.6 Statistical analysis

Mann-Whitney *U*-test (left-sided) is used for comparing the control group (either joints from healthy volunteers or non-inflamed joint from the same subject) with inflamed joints. Spearman’s rank correlation is used when comparing the PD grading with PA quantification.

## 3 Results

### 3.1 Subject characteristics

7 healthy volunteers and 10 RA patients were included in the study. All subjects had Caucasian skin. The characteristics of these subjects are shown in Table 1. The RA patients had a mean disease duration of 117 months (range 5–133), all were positive for rheumatoid factors and 7 were positive for anti-cyclic-citrullinated protein antibody (anti-CCP), and the mean C-reactive protein (CRP) levels prior to the measurement were 6.3 (SD 5.6).

**Table 1:** Subject characteristics.

Characteristic	Healthy volunteers	RA patients
	(N = 7)	(N = 10)
Age: mean (range)	56 (49–62)	63 (49–80)
Gender (% female)	43%	50%

Values are the subject’s mean (standard deviation, SD) or (range).

### 3.2 Photoacoustic/ultrasound imaging

**Fig. 2:**
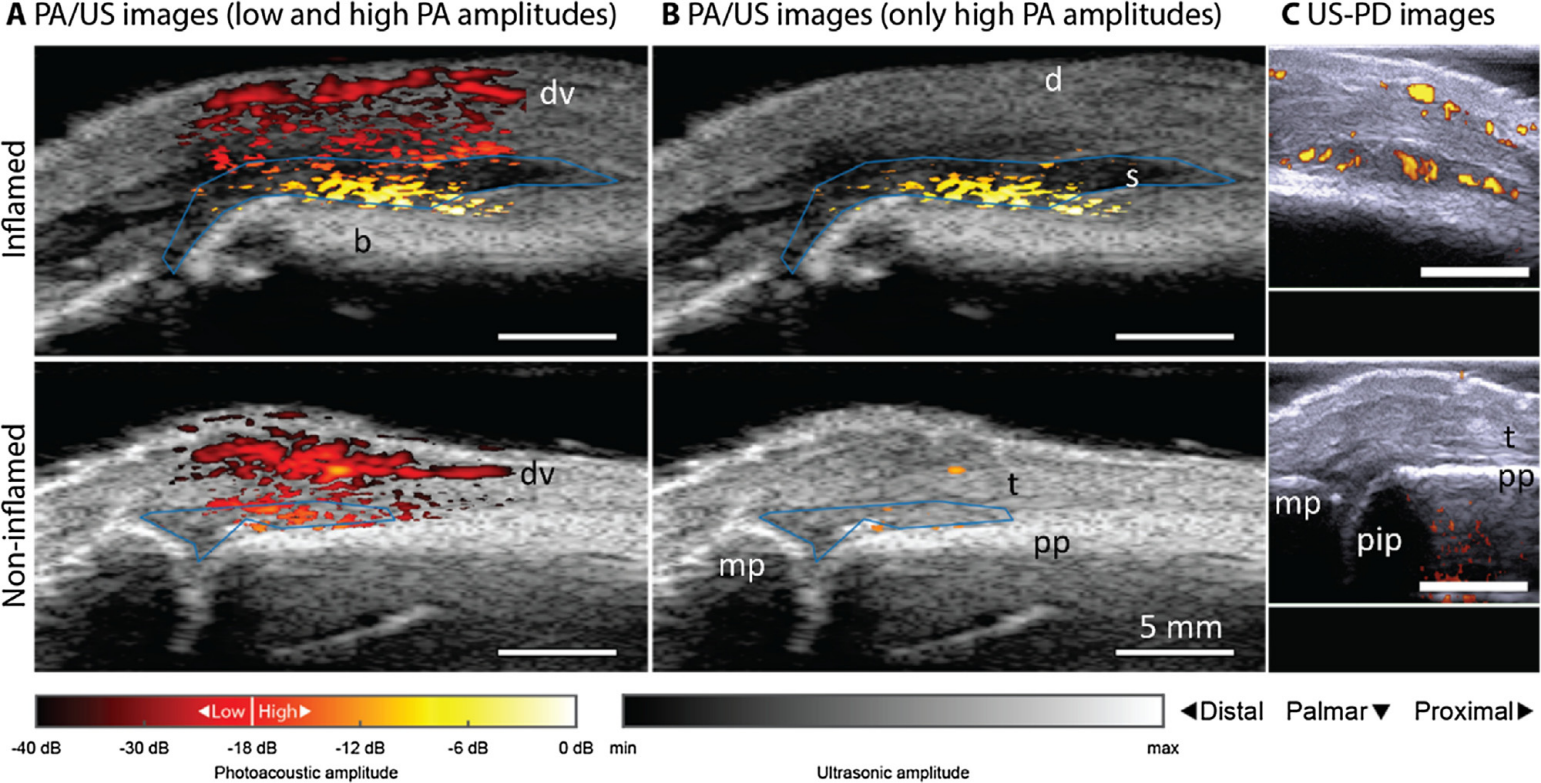
PA/US and US/PD images of an inflamed (upper row) and non-inflamed contra-lateral joint (bottom row) of an RA patient. PA/US images in (A) show a difference in color between inflamed and non-inflamed corresponding to an increase in amplitude levels. When discarding low PA amplitudes in (B), only features in the inflamed joint are visible. Corresponding US-PD images are shown in (C). The blue line in the PA/US images indicates the ROI used for quantification of PA features in the synovial space. The 0 dB level is the maximum PA amplitude from the inflamed joint. d = dermis; dv = dorsal vein; pp = proximal phalanx; pip = proximal interphalangeal joint; mp = middle phalanx; s = synovium; t = extensor tendon.

Fig. 2 depicts examples of fluence corrected PA/US and US-PD images for an inflamed joint and the contra-lateral non-inflamed joint of an RA patient. The reconstructed PA signals are shown ranging from dark red (low signal amplitudes, starting at −40 dB) to light yellow (high/abnormal signal amplitudes, up to 0 dB); the data is overlaid on the grayscale US b-mode image. The PA images in Fig. 2A show a superficial blood vessel in both the inflamed and non-inflamed joint, with additional PA features underneath, above the bone surface. Larger amplitudes and more confluent features are recorded for the inflamed joint, as can be further observed in Fig. 2B where only high amplitudes (18 dB dynamic range) are plotted. With this threshold, almost no PA features are visible for the non-inflamed joint.

### 3.3 Quantification of PA and US-PD imaging

**Fig. 3:**
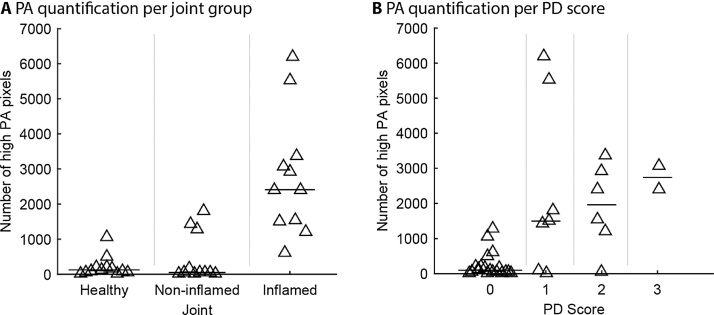
PA quantification with (A) comparing the number of high PA pixels for each joint group and (B) comparing the same quantification for discrete PD score (0, 1, 2 or 3, offset on the x-axis is to visualize individual markers); Spearman’s ρ = 0.64 (95% CI: 0.42, 0.79), p < 0.001. One triangle represents one joint and horizontal bar is median of one group.

The numbers of high amplitude PA pixels (such as those visible in Fig. 2B) were computed for inflamed and non-inflamed joints, and of joints from healthy volunteers. The result (Fig. 3A) indicates a larger number of high amplitude PA pixels for inflamed joints, compared to healthy and non-inflamed joints. In addition, an alternative quantification method for PAI also shows a larger value for inflamed joints: the mean (non-compressed) pressure amplitude of PA features (Table 2). Both quantification methods show 4 to 10-fold increased counts (p < 0.001) when comparing inflamed joints with those from control groups. Note also that the fingers are swollen: the size of the ROI as drawn on the grayscale US images is significantly larger in inflamed joints compared to healthy (p < 0.001) and compared to non-inflamed joints (p < 0.05). Grading of US-PD images shows a strong agreement (ρ = 0.64, 95% CI: 0.42, 0.79, p < 0.001) of the PA pixel count with the consensus PD score assigned to the images by two rheumatologists (Fig. 3B and Table 3).

**Table 2:** PD score, PA quantification and hypertrophic area (ROI size).

Parameter	Healthy	Non-inflamed	Inflamed
	(N = 12)	(N = 11)	(N = 11)
PD score	0.1 (0.3)***	0.5 (0.7)**	1.7 (0.9)
Number of high PA pixels	225 (299)***	444 (694)***	2792 (1742)
Mean PA amplitude	13.2 (4.4)***	14.9 (11.7)***	56.7 (36.0)
ROI size (pixels)	4540 (1318)***	7900 (3690)*	12468 (4554)

Quantification values: mean (standard deviation). Rank test p-values for testing inflamed joints versus either of the control groups (healthy or non-inflamed): ***p < 0.001, **p < 0.01 or *p < 0.05.

**Table 3:** PD score versus other parameters.

Parameter	PD-0	PD-1	PD-2	PD-3
	(N = 19)	(N = 7)	(N = 6)	(N = 2)
Number of High PA pixels	252 (367)	2368 (2494)	1909 (1219)	2741 (472)
Mean PA amplitude	12.2 (4.1)	43.8 (39.8)	50.1 (38.8)	53.6 (6.5)
ROI size (pixels)	5263 (2115)	11075 (5265)	12162 (3868)	14013 (4445)

Quantification values: mean (1σ).

**Fig. 4:**
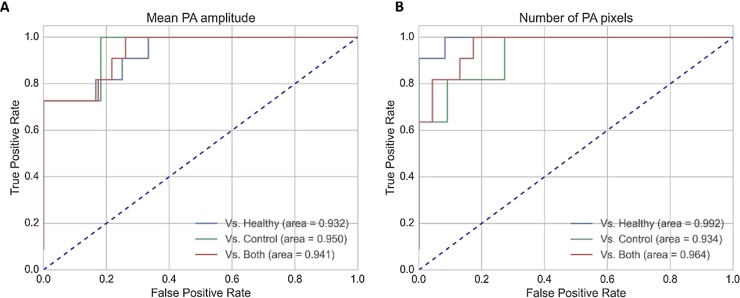
Receiver Operating Characteristics (ROCs) for the mean PA amplitude (A) and the number of high PA pixels exceeding −18 dB (B) within the regions of interest. Separate comparisons and areas under the curve are given of inflamed joints with joints in healthy subjects (‘healthy’) and contralateral joints in patients (‘control’).

To obtain an early impression on the diagnostic accuracy of the method, Receiver Operating Characteristics have been constructed for the mean PA amplitude in the regions of interest, and the number of high amplitude PA pixels, given in Fig. 4A and B, respectively. Separate curves and areas under the curve are given for inflamed joints vs. non-inflamed contralateral joints in patients, and vs. joints in healthy subjects.

## 4 Discussion

We found that PAI – in the first study with a handheld combined photoacoustic probe – was sensitive to clinically evident synovitis as demonstrated by the significant difference in PA features between inflamed and control joints. In addition, the PA quantification agreed well with the corresponding semi-quantitative PD scores. The ROCs and areas under the curve reveal a good separation of photoacoustic image characteristics between inflamed and non-inflamed joints. This observation must be treated with care, because of the small size of the study and the methodological limitations discussed below. Nevertheless, the results do encourage further research in photoacoustic imaging of early inflammations.

Hyper-vascularization and angiogenesis are hallmarks of rheumatoid arthritis and are markers for imaging with US-PD, as the increase in blood flow is detectable using ultrasound flow imaging. In joints that are close to the skin, the increase in vascularity is an attractive target for PAI. It should be realized, that US-PD and PAI do not provide an identical representation of vascularity, synovial or otherwise. On one hand, US-PD is expected to highlight larger feeding vessels and, theoretically, the movement of other structures such as villous synovial folds within the hypertrophic region. On the other hand, PAI is expected to be particularly sensitive to increased blood volume in smaller vasculature within the synovial membrane. PAI typically works best for small vessels, networked mostly parallel to the probe; US-PD rather visualizes large vessels, angled to the probe. The unique photoacoustic probe that we used in this study is sensitive to vessels, or vascular networks of 0.2 mm in size and larger. Interestingly, the appearance of synovitis in PAI is quite similar for all the clinically inflamed joints that were imaged in this study − unlike that of the US-PD representation, which varied considerably.

These fundamental differences between PAI and US-PD may help explain the variation between the PD score and the PAI quantification (Fig. 3B). There are a few data points that fall outside the ‘natural’ spread: Fig. 3B shows two grade 1 joints with a very high photoacoustic signal and three grade 1–2 joints that hardly show a PAI signal. The former (“too high PAI signal”) may originate from a different source, as the shape of these corresponding structures was decidedly different from the regularly seen representation of the synovium in PAI. The latter offsets (“too low PAI signal”) may in fact be due to false positive PD scoring a result of artefacts: notes from one of the two blinded examiners confirm this possibility.

While this work provides evidence of PAI detecting synovitis, there exist a few methodological limitations to this study. For one, the selection of patients took place approximately a week before the PA examination. This may explain partly the variance in the PA quantification of inflamed joints (Fig. 3A), as some patients’ synovitis subsided after selection, but were still included in the inflamed group. In addition, US-PD is hard to standardize, which may have caused the PD artefacts explained earlier. Also, the researcher in charge of drawing the ROIs was not blinded to the joint inflammation, which may have biased the interpretation. This issue was moderated however, since the ROI was drawn on the US image without showing the PA overlay. A technical limitation of the system was the inability to co-acquire high-quality b-mode and PA images. The short delay between both may have resulted in inaccuracy due to accidental movement of the finger. This limitation of our setup will be solved in a future version, leading to almost simultaneous acquisition of PA and b-mode US images. Despite these limitations this study shows positive and highly significant findings in PAI. Fluence correction appeared to be necessary in our analysis. Variations of the applied exponential fluence decay rate in a realistic interval around the assumed value of 1/mm, had no critical influence on the outcome of our analysis.

This is the first clinical study with a compact and fully integrated PA/US imaging probe. It means an important step from existing PAI systems, where sizable and costly external lasers are used, towards practical use in clinical settings. Furthermore, our system relies on a near infrared (NIR) light source at 808 nm, in contrast to previous studies, which used visible light of 580 nm [44]. While haemoglobin absorbs less NIR light than it does in the visible range, light attenuation in the surrounding tissue is also lower. This means that with NIR light the PA outcome depends less on the exact tissue composition. In addition, absorption by superficial structures would be much more pronounced with visible light, for instance in the melanin layer and of regular vessels. Absorption like this is known to cause pronounced clutter when these PA signals also travel down and reflect on lower structures.

Previous studies showed that linear array-based systems such as used in this study are susceptible to clutter and reflection artefacts [45]. Future studies should therefore include clutter reduction and artefact removal [46], [47]. We were able to reject the possibility of most types of artefacts by moving the illumination position in relation to the finger – for most types of artefacts the appearance of PA features would move in relation to the US image [48], but this did not happen in the cases investigated here. However, clutter may have caused the baseline PA signal as can be seen in Fig. 2, and also some of the outlying data points in Fig. 3.

Future applications of PAI to synovitis can take advantage of its multi-spectral imaging capabilities, allowing the estimation of the oxygenation saturation (sO_2_) of the synovium. Multi-spectral PAI is expected to improve the specificity of the technique. Targeted PA contrast agents [49] with specific spectral signature linked to molecular markers also deserve investigation, as they could provide information about inflammation similar to for example positron emission tomography. The next prototype of our probe includes diode lasers of various wavelengths for this purpose. This prototype merits further investigation of subclinical synovitis in a larger patient population, and its predictive value for a disease flare. In addition, comparison with MRI angiography will allow a closer look at which specific vascular structures are depicted by PAI. A current limitation of the handheld probe is its low penetration depth (15 mm) compared to other PAI systems, which means future applications will likely focus on peripheral joints that are close to the skin.

## 5 Conclusion

PAI is a unique modality due to its optical imaging contrast in combination with ultrasound-based resolution. We have shown that PAI with a handheld probe can detect clinically evident synovitis, which is a first step toward the application of PAI for diagnosis and monitoring of inflammation in peripheral joints. These results provide a basis for further research to investigate the potential benefits of PAI over other modalities.

## Contributions

All authors took part in the conception and design of the study. PJB, KD and HJBM performed the measurements. PJB processed and analysed the data and wrote the manuscript draft. HJBM took part in grading the US-PD images. Each author took part in editing the manuscript, read and approved its final version.

## Patient consent

Written informed consent was obtained prior to inclusion.

## Funding

The research leading to these results has received funding from the European Commission’s Seventh Framework Programme (FP7/2007-2013) under grant agreement no. 318067, and the European H2020 program under grant agreement no. 731771.

## Ethics approval

The ethical committee METC Twente gave its approval of the study protocol.

## Conflict of interest

The authors declare that there are no conflicts of interest.
